# Preparation and neutralization efficacy of IgY antibodies raised against *Deinagkistrodon acutus* venom

**DOI:** 10.1186/s40409-017-0112-0

**Published:** 2017-04-04

**Authors:** Jinhua Liu, Qiyi He, Wenwen Wang, Bin Zhou, Bo Li, Yingfeng Zhang, Cong Luo, Diancheng Chen, Jia Tang, Xiaodong Yu

**Affiliations:** 1 Animal Toxin Group, Chongqing Key Laboratory of Animal Biology, Chongqing Engineering Research Center of Bioactive Substances, Engineering Research Center of Active Substances and Biotechnology, Ministry of Education, College of Life Science, Chongqing, 401331 China; 2 Library, Chongqing Normal University, Chongqing, 401331 China

**Keywords:** IgY antibody, Egg yolk, Snake venom, Snakebite, *Deinagkistrodon acutus*, Venom neutralization

## Abstract

**Background:**

The five-paced pit viper (*Deinagkistrodon acutus*), endemic to China and northern Vietnam, is responsible for most snakebites in the Chinese territory. Antivenom produced from horses is the main treatment for snakebites, but it may cause numerous clinical side effects and have other disadvantages involved in their production such as the welfare of animals. The present study was conducted aiming to develop an alternative antibody (IgY) from the egg yolk of leghorn chickens immunized with snake venom.

**Methods:**

IgY from the egg yolk of white leghorn chickens previously immunized intramuscularly with *D. acutus* venom was extracted by water, precipitated by ammonium sulfate and purified by affinity chromatographic system. IgY was identified by SDS-PAGE, ELISA and Western blot. Finally, IgY neutralization assays to test its efficacy against hemorrhagic, edema-forming and myotoxic activities of *D. acutus* venom were conducted on mice.

**Results:**

For the first time, IgY antibodies against *D. acutus* venom were raised successfully in egg yolk of chickens injected with *D. acutus* venom multiple times. By three steps, including caprylic acid extraction, ammonium sulfate precipitation and affinity chromatography, IgY antibodies were isolated and purified from egg yolk, which exhibited a single protein band on SDS-PAGE and two bands (about 65 kDa and 35 kDa, respectively) under reducing conditions, and presented a high titer (1:40,000) tested by ELISA. Immunoblot analysis confirmed that these IgY were polyclonal antibodies since they bound to components of *D. acutus* venom. Furthermore, immunodiffusion assay showed that anti-*D. acutus* venom IgY cross-reacted with the venoms of *Trimeresurus albolabris* and *D. saxatilis* Emelianov, but did not react to the venoms of *Bungarus multicinctus* and *Naja atra*. In the neutralizing lethal assay, the median effective dose of anti-*D. acutus* venom IgY was 14.14 mg/kg of mouse body weight under the challenge dose (3 LD_50_ of *D. acutus* venom). In neutralizing the hemorrhagic, edema-forming and myotoxic activities of *D. acutus* venom, IgY showed the characteristic dose-dependent neutralization effects against all these toxic activities of *D. acutus* venom.

**Conclusion:**

Anti-*D. acutus* venom IgY antibodies with high purity and titer were for the first time raised successfully in egg yolk of chickens immunized with *D. acutus* venom. They were effective in neutralizing the lethal effects, and the hemorrhagic, edema-forming and myotoxic acitivities of *D. acutus* venom. IgY could be an effective source to develop a treatment against snake bites in humans or animals in the future.

## Background

Snake envenomation is an important public health issue in the world. There are more than 420 species of venomous snakes, which are widely distributed throughout the globe (including oceans), except for a few islands, frozen environments and high-altitude regions. These animals provokes 5.4 million bites, about 2.5 million of envenomation cases and over 0.125 million of deaths annually. As is well known, the highest burden of snakebites is found in South Asia, Southeast Asia and sub-Saharan Africa [1–4]. In addition, the burden of snakebites is heavy in China, where there are over 50 species of venomous snakes. One species, the five-paced pit viper (*Deinagkistrodon acutus*), is responsible for most snakebites in the country. It is a large sized and highly venomous snake, found from the southern provinces of China (including Taiwan) to northern Vietnam [5, 6].

The intravenous administration of antivenom is the mainstay treatment for envenomated victims. The conventional antivenom is generally produced from the blood of the large animals, frequently horses or sheep, immunized with the venom of a single snake or mixed venoms obtained from several animals to eliminate the intraspecific diversification [7]. Nevertheless, the serum used to treat snakebite victims may cause various side effects, such as anaphylactic shock, pyrogen reaction and serum sickness, which are mainly caused by the nonspecific proteins found in commercially available antivenoms [8–11]. Moreover, frequent immunization and bleeding during antivenom production may cause great distress to the employed animals. In addition, the production of sufficient pure serum antibodies against snake venom may require more investment and time, which may be conflicting with interests of serum manufacturers, especially in developing countries. Thus, it is necessary to find an alternative way of antibody creation, which would be safer, cheaper, short-cycle and non-invasive.

In recent years, eggs of chicken immunized with antigens have been recognized as a good source of IgY antibodies [12–14]. So far, many research results about snake antivenom IgY have demonstrated that IgY in eggs of chicken is an excellent alternative to the conventional antivenom produced in large mammals [15, 16]. The main advantages of IgY include:

- collecting eggs instead of bleeding animal meets the animal welfare requirement;
- the cost of keeping hens is lower than that of raising horses or sheep;
- IgY antibodies do not cross-react with Fc receptors, thereby reducing the cause of false positive results in immunological assays;
- IgY, like mammalian IgG, is a reasonable stable protein over time;
- the yield of IgY is large and its production may be readily scalable [17–20].

In this study, hens were hyperimmunized with low doses of *D. acutus* venom to produce specific antibodies. IgY was extracted from egg yolk and further purified by affinity chromatography. Subsequently, we assessed and examined the purity, the binding specificity, the titer and the neutralization efficiency of IgY. All the results will provide a basis for developing IgY into a clinical agent in the future.

## Methods

### Reagent and supplies

Freund’s complete adjuvant (FCA), Freund’s incomplete adjuvant (FIA), rabbit anti-IgY peroxidase conjugate, Immobilon®-P (polyvinylidene difluoride, PVDF), a transfer membrane with pore size of 0.45 μm, 3^1^,3^1^,5^1^,5^1^-tetramethylbenzidine (TMB), nonfat dry milk and horseradish peroxidase-conjugated rabbit anti-chicken IgY were purchased from Sigma (USA). Polystyrene ELISA plates were purchased from Corning (USA). Amicon ultra-15 centrifugal filter devices were obtained from Millipore (USA). NHS activated Sepharose 4 FF were purchased from GE Healthcare (UK). Protein molecular weight markers were purchased from Takara (Japan). All the other reagents were of analytical grade.

### Venom

Venom was obtained from *D. acutus* captured at Wulingshan in Chongqing, China. Venom was lyophilized in a ModulyoD-230 freeze dryer (Thermo Scientific) and stored at −20 °C until use.

### Animal

Seventeen-week-old white leghorn hens weighing 1.5 kg each, purchased from a local poultry farm, in good health and laying conditions (laying 5 to 6 eggs per week) were used for the production of IgY against snake venom. They were kept in individual cages with standard food and water. Kunming mice (18–20 g) were purchased from experimental animal center of Third Military Medical University. Mice were kept in plastic boxes at five per cage, in a room maintained at 20–23 °C on a 12/12-h light/dark cycle with food and water *ad libitum*.

### Immunization schedule

Based on the LD_50_ of *D. acutus* venom for mice (about 2.93 mg/kg, intraperitoneally), the LD_50_ of *D. acutus* venom for laying hens was calculated to be 0.72 mg/kg. Each hen was immunized intramuscularly at multiple sites in the breast region with 0.5 mL saline (containing 0.29 mg snake venom) emulsified with an equal volume of FCA. On the 14^th^, 35^th^ and 56^th^ day after the first immunization, booster doses were administered with 0.5 mL saline (containing 0.58 mg, 1.17 mg and 1.17 mg snake venom, respectively) emulsified with an equal volume of FIA. Serum was collected weekly from the first immunization, but after the 10^th^ week serum was collected every 2 weeks. Eggs began to be collected daily before the first immunization and the collecting eggs sustained for 24 weeks after the first immunization. The control group of hens was immunized intramuscularly with 0.5 mL saline. Serum was stored at −20 °C and eggs at 4 °C until use.

### Extracting antibody from egg yolk

Extraction of IgY from preimmunized and hyperimmunized eggs was performed according to our previous method with minor modifications [21]. Briefly, the egg shell was cracked and the yolk was separated from the egg white. The yolk contents was diluted 7.5-fold with deionized water and homogenized by stirring vigorously for 30 min on magnetic stirrer. The resulting homogenate was further diluted 2-fold with 0.04 M acetate buffer (pH 5.0, containing 0.06 M NaCl) and again homogenized for 30 min while adding caprylic acid up to final concentration of 1%. The preparation was placed at room temperature for 4 h. The clear supernatant, the water-soluble fraction (WSF), was siphoned out and centrifuged at 10,000 rpm for 10 min at 4 °C. The IgY in water-soluble fraction was precipitated out with 45% ammonium sulfate. The salt pellets were dissolved in phosphate buffered saline (PBS, pH 7.4) and dialyzed against PBS. Finally, the partially purified antibody preparation (crude extract) was subjected to affinity chromatography.

### Affinity purification

The venom affinity column in chromatographic system (ÄKTA purifier 100, GE) was prepared as follows: in brief, NHS activated Sepharose 4FF were coupled with whole venom of *D. acutus* dissolved in coupling buffer (0.2 M NaHCO_3_, pH 8.3, containing 0.5 M NaCl). Unreacted groups on the Sepharose were blocked with 0.5 M ethanolamine buffer (pH 8.3, containing 0.5 M NaCl). Finally, the Sepharose was washed with Tris–HCl buffer (0.05 M, pH 9.0) and then acetate buffer (0.05 M, pH 5.0). The Sepharose was packed into a column that was equilibrated with PBS buffer (0.01 M, pH 7.4). The crude extract (ant-*D. acutus* IgY, 20 mg/mL) was loaded into the affinity column, and the column was washed with PBS buffer (0.01 M, pH 7.4) to remove the unabsorbed proteins. The bound antibodies were eluted with glycine-HCl buffer (0.1 M, pH 2.3), and pooled, concentrated and desalted through Amicon Ultra-15 centrifugal filter devices, then stored at 4 °C.

### Protein concentration

Protein concentration was measured as described by Lowry et al. [22].

### SDS-PAGE

The purity and molecular weight of the IgY were analyzed on 12% sodium dodecyl sulfate-polyacrylamide gel electrophoresis (SDS-PAGE), according to the method of Laemmli [23].

### Western blot

It was carried out according to the procedure of Towbin et al. [24] with minor modifications. *D. acutus* venom (50 μg) were separated on 15% non-reducing SDS-PAGE, and then electroblotted onto PVDF membrane that was treated with methanol before. The unreacted sites on membrane were blocked with a blocking solution (5% nonfat milk in PBS buffer, pH 7.4 and containing 0.05% Tween-20) at room temperature for 2 h on a horizontal shaker, then incubated with the affinity-purified anti-*D. acutus* IgY for 1 h at room temperature. Next, the blot was washed five times with rinse buffer and incubated with horseradish peroxidase-conjugated rabbit anti-chicken IgY (1: 5000). Finally, the blots were washed and specific blots of venom proteins bound to IgY were visualized using peroxidase chromogenic substrate solution TMB.

### Immunodiffusion assay

The specificity of IgY against snake venom components was demonstrated with agar diffusion test (1% agarose) described by Ouchterlony [25]. *D. acutus* venom was added into the center well in an agar plate, the different concentrations of anti-*D. acutus* IgY antibodies were loaded into the peripheral wells, and then the plate was incubated for 24 h at 37 °C to observe the antigen-antibody reactive lines. In addition, the cross reactivity between anti-*D. acutus* IgY and four other snake venoms (*Trimeresurus albolabris, A. saxatilis, Bungarus multicinctus* and *Naja atra* from China) was also evaluated by this test.

### ELISA assay

ELISA was used to assess the activities of anti-*D. acutus* IgY antibodies in serum and the yolk [21]. The microplates were coated with 1 μg native *D. acutus* venom in 100 μL coating buffer (0.05 M carbonate bicarbonate, pH 9.6) for 14 h at 4 °C and the wells were washed with rinse buffer (PBS-0.05% Tween 20, pH 7.4). Then, the unbound sites were blocked for 1.5 h at 37 °C with 200 μL blocking buffer (rinse buffer plus 5% nonfat dry milk). The coated wells were washed again, and then were added to the serum or the yolk diluted in dilution buffer (PBS plus 1% nonfat dry milk) and incubated 1 h at 37 °C. After the wells were washed three times, they were added to the rabbit anti-IgY-peroxidase dilution (1:5000) and incubated for 1 h at 37 °C. After the wells were washed once again, 200 μL of TMB was added and incubated at room temperature for 20 min. The reactions were terminated with 50 μL of 2 M sulfuric acid. Absorbance was recorded at 450 nm using ELISA plate reader. Results determination: a positive sample and a negative sample need to give a ratio of at least 2.1 (that is, OD of the positive divided by OD of the negative. P/N > 2.1).

### Neutralization of venom lethality by IgY

The ability of IgY to neutralize lethality of *D. acutus* venom was assessed by an in vivo neutralization test. Various amounts of the purified antivenom IgY (50 μg, 100 μg, 200 μg, 400 μg, 800 μg and 1600 μg, respectively) were mixed with the challenge dose (3 LD_50_ dose of *D. acutus* venom) and incubated for 30 min at 37 °C, centrifuged for 5 min, then 20 μL of the supernatant was injected intraperitoneally into groups of 10 mice. Control mice received the same amount of venom mixed with the normal yolk. The deaths were recorded over 72 h. The ED_50_ was calculated according to the recommendation of WHO [26] and expressed as mg IgY/kg body weight.

### Neutralization of hemorrhagic activity by IgY

Hemorrhagic activity was quantitatively determined according to the method of Gutiérrez et al. [27]. Various amounts of venom (0, 5 μg, 10 μg, 15 μg, 20 μg and 25 μg) diluted in 0.1 mL physiological saline (0.9% w/v) were injected subcutaneously into the dorsal skin of groups of four mice. Two hours later, they were sacrificed and their dorsal skin was removed, and the diameter of the hemorrhagic spot was measured. Physiological saline was utilized as negative control. Diameters were calculated and the minimum hemorrhagic dose (MHD) was defined as the dose of venom that induced a lesion of 10 mm of diameter 2 h after injection. The challenge doses of venom (3 MHD) was incubated with various amounts of IgY antibodies (0, 500 μg, 1000 μg, 1500 μg, 2500 μg, and 3500 μg) for 30 min at 37 °C, centrifuged for 5 min. Then, 10 μL of the supernatant was subcutaneously injected into the dorsal skin of groups of ten mice. Control group received normal yolk (the same amount of used for venom). Hemorrhage was expressed as a percentage, taking as 100% the diameter of the lesions induced by inoculating 10 MHD of venom alone. Results were plotted and the ED_50_ was defined as the ratio of IgY/venom that decreased the activity by 50%.

### Neutralization of edema-forming activity by IgY

Edema-forming activity was quantitatively determined according to the method of Van Dong et al. [28]. There are groups of 4 mice injected subcutaneously, in the right footpad, with 20 μL of various amounts of venom (0.1 μg, 0.5 μg, 1 μg, 1.5 μg and 2.0 μg) dissolved in physiological saline(0.9% w/v), and in the left footpad received 20 μL of the physiological saline. The weight of each foot was determined 3 h after injection with an electrionic scale. The minimum edema-forming dose (MED) corresponds to the amount of venom that induces an increment of 30% in the weight of the foot envenomated for 3 h when compared with the physiological saline-injected foot. The challenge doses of venom (3 MED) was incubated with various amounts of the IgY antibodies (25 μg, 50 μg, 100 μg, 150 μg and 200 μg) for 30 min at 37 °C, centrifuged for 5 min, then 20 μL of the supernatant was injected subcutaneously into groups of 10 mice in the right footpad.

### Neutralization of myotoxic activity by IgY

Myotoxic activity was quantitatively determined according to the method of Rojas et al. [29]. Various amounts of venom(0 μg, 5 μg, 10 μg, 15 μg, 20 μg and 25 μg) dissolved in 50 μL of physiological saline (0.9% w/v) were injected intramuscularly (i.m.) into groups of 4 mice in the right gastrocnemius muscle. Control animals received 50 μL of the physiological saline. Three hours after injection, mice were bled from the orbital plexus, under CO_2_ anesthesia. Plasma creatine kinase (CK) activity was quantitated by the CK kit (Nanjing Jiancheng Corp. China). CK activity is expressed in units/L, 1 U defined as the amount of enzyme that produces 1 μmol of NADH under the conditions of the assay. The minimum myotoxic dose (MMD) corresponds to the amount of venom that induces an increment of plasma CK activity corresponding to four times the activity of mice injected with physiological saline alone. The challenge doses of venom (3 MMD) was incubated with various amounts of the IgY antibodies (0, 375 μg, 750 μg, 1500 μg and 3000 μg) for 45 min at 37 °C and centrifuged for 5 min. Then, 50 μL of the supernatant was injected subcutaneously into groups of ten mice in in the right gastrocnemius muscle.

### Statistical analysis

Statistical calculations were performed using GraphPad Prime 5.0. Results were presented as mean ± SEM values. The ANOVA was used to test differences among groups. The differences between the mean values were determined by the Student’s test and the differences were considered statistically significant at *p* < 0.05.

## Results

### Antibody response

**Fig. 1:**
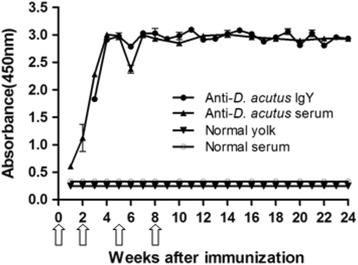
Primary and secondary antibody response in serum and egg yolk of hens immunized with *D. acutus* venom. The activity against *D. acutus* venom of serum and WSF was assessed by ELISA. The *white arrows* indicate immunization

Following primary immunization, the hens produced comparable antibody response that was detected in serum by day 8. Although the pre-booster response was low in the serum, after the first booster there was a sharp increase in antibody titer both in serum and egg yolk. Three weeks after primary immunization, transference of antibodies specific to venom from serum to egg yolk was observed. At the 4^th^ week, the antibody response both in serum and yolk reached the highest level and maintained thereafter at least for 20 weeks (Fig. 1).

### Extraction, purification and biochemical identification of IgY

**Fig 2:**
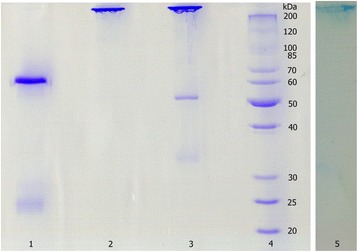
IgY samples from egg yolk analyzed by 12% SDS-PAGE and identified by Western blot. Lane 1 and lane 2: 10 μg of IgY after affinity chromatography (reducing and non-reducing conditions, respectively). Lane 3: 15 μg of IgY’s crude extract. Lane 4: molecular weight marker. Lane 5: identification of IgY with HRP-rabbit anti-chicken IgY by Western blot

The partially purified antibody preparation (crude extract) was obtained from the egg yolk by the water-soluble extraction method; then, it was fractioned by affinity chromatography into two peaks with strong IgY activity. They exhibited only one band (about 182 kDa) on SDS-PAGE under non-reducing conditions. However, under reducing conditions, they presented two bands, a heavy chain of IgY of about 66 kDa and a light chain of IgY of about 25 kDa (Fig. 2). Western blot showed that the purified sample could be recognized by HRP-rabbit anti-chicken IgY (Fig. 2). The average recovery of venom-specific IgY from 131 eggs was about 18.47% (Table 1). The venom-specific activities of IgY on different fractions – including water-soluble fraction (WSF), salting-out fraction (SOF) and thiophilic-chromatography fraction (TCF) – were compared by ELISA. The results showed that the titer of TCF is twofold higher than that of SOF, and 16 times higher when compared with to WSF. Obviously, the three purification steps used resulted in the enrichment of venom-specific IgY.

**Table 1:** Recovery ratio of proteins and titer of IgY fractions

Fractions	Titer of IgY by ELISA (× 10^4^)	Yolk proteins in 131 eggs (g)	Recovery ratio of proteins (%)
WSF	3.3	31.83	100
SOF	25.7	12.59	39.56
TCF	51.3	5.88	18.47

*WSF* Water-soluble fraction, SOF Salting-out fraction, *TCF* Thiophilic-chromatography fraction

### Immunological identification of IgY’s crude extract

**Fig 3:**
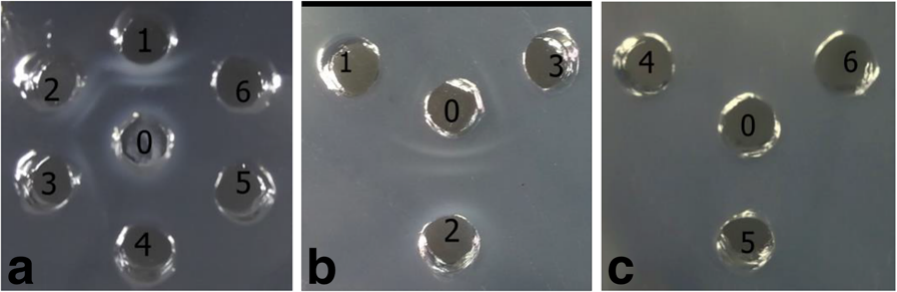
Immunological assessment of anti-*D. acutus* venom IgY against venom from five species of snakes found in China by immunodiffusion assay. **a** In the central well (0): *D. acutus* venom (30 μg); in the peripheral wells: (1) anti-*D. acutus* IgY crude extract (1200 μg); (2–5) the serial dilutions (1:2, 1:4, 1:8, and 1:16, respectively) of the crude extract; (6) physiological saline. **b** and **c** In the central well (0) anti-*D. acutus* IgY crude extract (1200 μg) whereas the the peripheral wells contained 30 μg of the following: (1) *T. albolabris* venom; (2) *D. acutus* venom; (3) *D. saxatilis* Emelianov venom; (4) *B. multicinctus* venom; (5) *N. atra* venom; and (6) physiological saline

Thirty micrograms of *D. acutus* venom was added to the central well, and the serial dilutions of the crude extract were added to the peripheral wells, respectively. The white precipitation lines occurred between the central well and each peripheral well indicated the immunological activities of IgY (Fig. 3). The titer of the crude extract by immunodiffusion was about 1/8. About 1200 μg of crude extract was added to the central well and the five different snake venoms (stored in our laboratory) were placed in the peripheral wells. The results indicated that the venoms of three species (*T. albolabris*, *D. acutus* and *D. saxatilis* in China) of Viperdae could react with the anti-*D acutus* IgY, but the venoms of two species (*B. multicinctus* and *N. atra* in China) of Elapidae could not (Fig. 3).

### Evaluation of purified IgY titer

**Fig 4:**
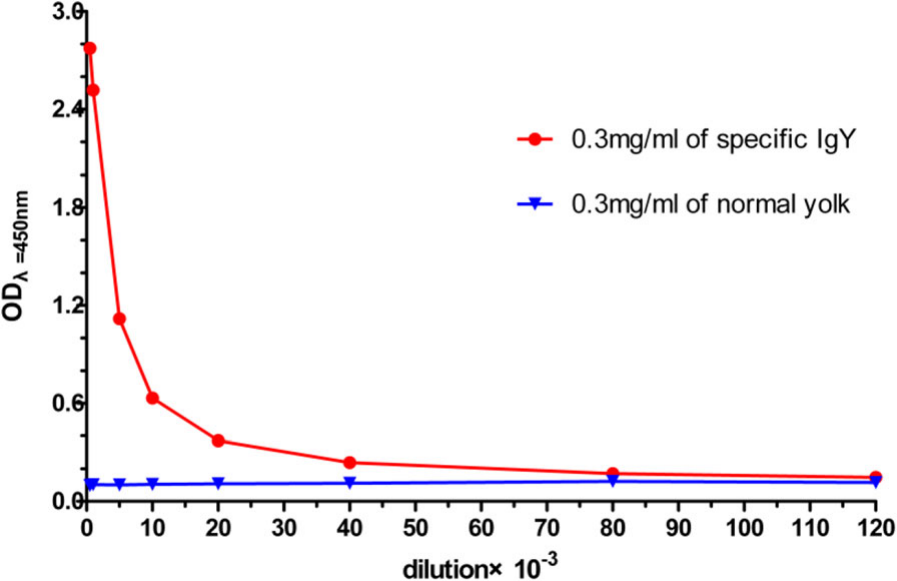
Titer profile of IgY raised against *D. acutus* venom by ELISA. When the value of P/N is bigger than 2.1, the sample was positive (*n* = 3)

According to the results of ELISA, the titer of specific anti-*D. acutus* venom IgY (0.3 mg/mL), which had been concentrated and desalted by ultrafiltration, was 1:40000 (Fig. 4).

### Antigen recognition repertoire of IgY

**Fig 5:**
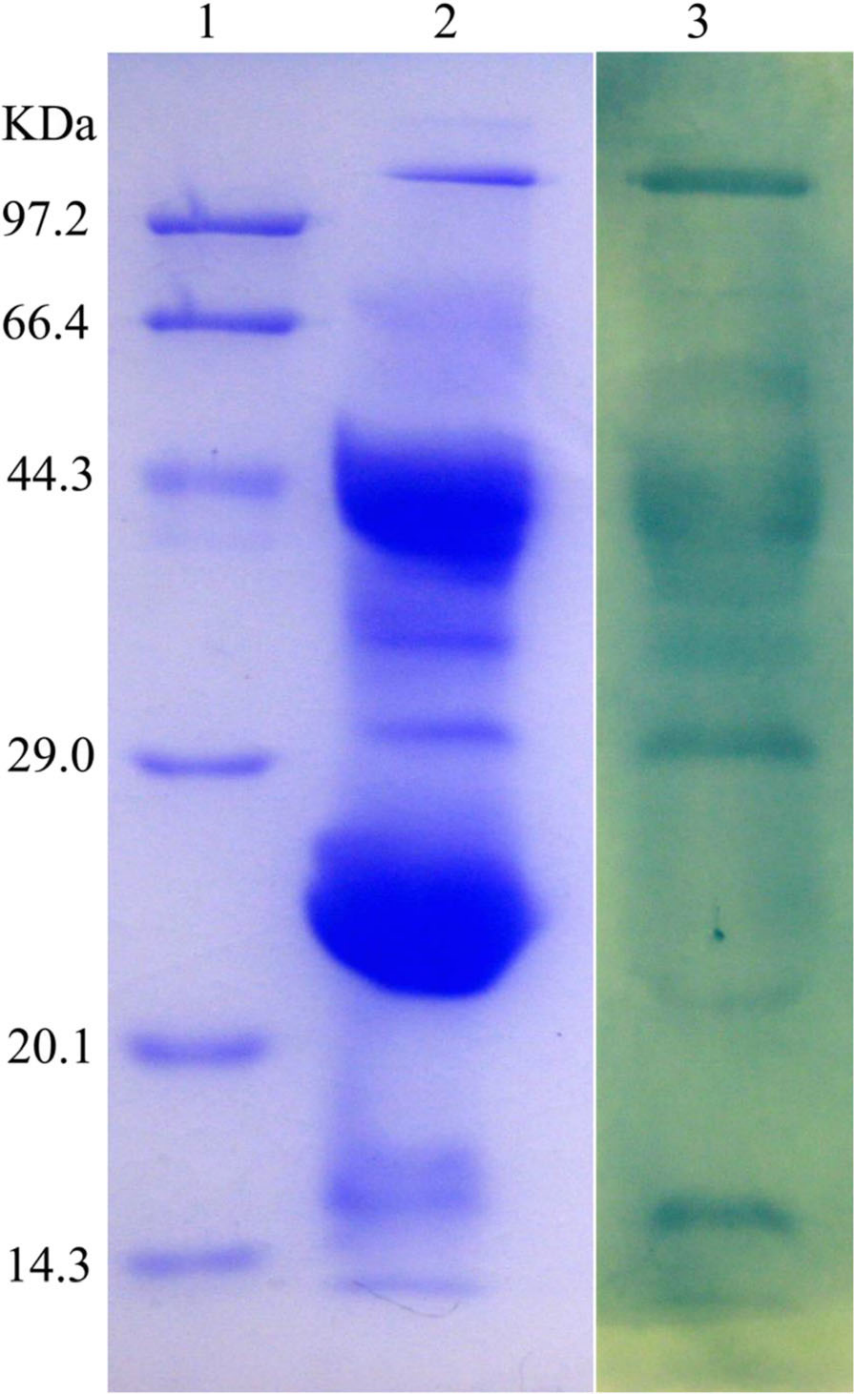
Antigen recognition repertoire of anti-*D. acutus* venom IgY by Western blot. Lane 1: Molecular weight marker on SDS-PAGE. Lane 2: *D. acutus* venom (50 μg) on SDS-PAGE. Lane 3: the protein components of *D. acutus venom* (50 μg) on Western blot were recognized by IgY that was recognized by HRP-rabbit anti-chicken IgY

Western blot analysis was carried out by using anti-*D. acutus* venom IgY as the first antibody and using HRP-rabbit anti-chicken IgY as the second antibody. The results obtained demonstrated that not all protein components of *D. acutus* venom were recognized by IgY (Fig. 5).

### Neutralization studies of anti-*D. acutus* venom IgY

Anti-*D. acutus* venom IgY that was in incubation with the venom prior to injection was capable of neutralizing the toxic components of venom. There was an increase in the survival rate of mice with increase in antivenom IgY. The proportion of 40 mg of IgY/kg of mouse body weight could produce 100% protection against a 3 LD_50_ dose (3 × 2.93 mg/kg) of venom and the value of ED_50_ was 14.14 mg IgY/kg. There were no survivals in the control group.

**Fig 6:**
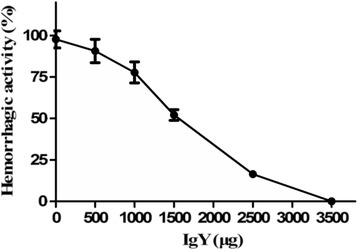
Neutralization of hemorrhagic effects of *D. acutus* venom by IgY (mean ± SD, *n* = 4). Mixtures containing a constant amount of venom and various dilutions of IgY were incubated at 37 °C for 30 min. Afterwards, aliquots of the mixtures (10 μL containing 3 MHD of venom) were injected subcutaneously into the dorsal skin of mice, whereas control group received the same amount of venom with normal yolk. Hemorrhage was expressed as a percentage, taking as 100% the diameter of the lesions induced by inoculating 10 MHD of venom alone

Concerning hemorrhagic activity, about 10 μg of *D. acutus* venom produced a hemorrhagic spot of 10 mm diameter (MHD). It was estimated that 350 μg of IgY was able to completely neutralize the hemorrhage induced by the challenge doses of venom (3 MHD), whereas 150 μg of IgY was able to neutralize about 50% of the hemorrhagic effects. Control mice injected with physiological saline or IgY showed no hemorrhage, respectively (Fig. 6).

**Fig 7:**
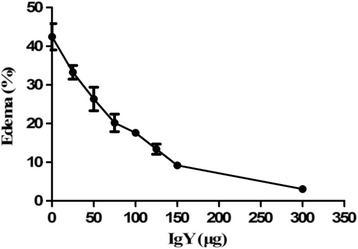
Neutralization of edema-forming activity of *D. acutus* venom by IgY (mean ± SD, *n* = 4). Mixtures containing a constant amount of venom and various dilutions of IgY were incubated at 37 °C for 30 min. Afterwards, aliquots of the mixtures (20 μL containing three MED of venom) were injected into the right footpad of mice, whereas the left foot pad received 20 μL of physiological saline. The weight of both feet was estimated 3 h after injection with an electronic scale and edema was expressed as the percentage increment in weight of the right footpad when compared to the left one

Regarding the edema-forming activity, mice immunized with *D. acutus* venom showed increase in footpad weight. About 1.3 μg of *D. acutus* venom induced edema formation within 3 h, which is considered as MED value. When anti-*D. acutus* venom IgY was incubated with the challenge doses of venom (3 × MED) for 30 min prior to injection, IgY antibodies were capable of inhibiting edema-forming activity induced by venom in a dose-dependent manner (Fig. 7), and ED_50_ of IgY was about 124.68 μg.

**Fig 8:**
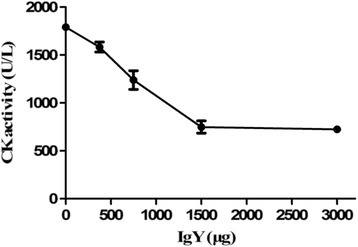
Neutralization of myotoxic activity of *D. acutus* venom by IgY (Mean ± SD, *n* = 4). Mixtures containing a constant amount of venom and various dilutions of IgY were incubated at 37 °C for 30 min. Then, aliquots of the mixtures (50 μL containing 3 MMD of venom) were injected into the right gastrocnemius muscle of mice, whereas control group received 50 μL of physiological saline. CK activity was expressed in U/L

In myotoxic activity, mice immunized with *D. acutus* venom showed increase in plasma CK activity. About 9.5 μg of *D. acutus* venom induced myotoxic activity, which is considered a MMD value. When anti-*D. acutus* venom IgY was incubated with the challenge doses of venom (3 × MMD) for 30 min prior to injection, IgY antibodies were capable of inhibiting CK activity induced by venom in a dose-dependent manner (Fig. 8), and ED_50_ of IgY was about 766.43 μg.

## Discussion

The venom of *D. acutus*, a snake endemic to China, possess proteins and peptides whose activity is mainly hemotoxic [28]. At the bite site, swelling, bruising, blistering and necrosis usually develop within a few minutes or hours and spread rapidly, sometimes the whole limb is affected [6]. The persistent bleeding from the fang marks and other previous partially healed wounds indicates coagulopathy, which is caused by the abundant metalloproteinases and serine proteases of *D. acutus* venom [5, 6]. Its toxicity and pathophysiologic effects can be completely reversed by specific antivenom, which is conventionally produced from blood of large animals (horses or sheep) immunized with snake venoms [8, 10]. As an alternative to the conventional antivenom with various side effects and the disadvantages of its preparation and production, antivenom IgY antibodies from egg yolk of chicken have considerable advantages [8, 16, 29]. Previous studies have shown that chicken immunized with snake venom produces IgY that may neutralize the toxic and lethal effects of venom and may serve to treat domestic animals affected by snakebites [16, 21, 30]. The current study for the first time described the creation of anti-*D. acutus* venom IgY antibodies in chicken egg yolk and assessed their efficacy in neutralizing the lethal effect and other activities of *D. acutus* venom.

The antibody production started in serum eight days after the first injection of *D. acutus* venom and in egg yolk at the 15^th^ day, increasing progressively along the immunization procedure, and attaining a plateau after the second booster, which was maintained thereafter (Fig. 1). This antibody response induced by *D. acutus* venom is for the first time obtained and is in good accordance with the antibody response of other snake venoms reported [21, 29, 30].

As shown by Duan et al. [21], three steps were chosen to extract and purify the specific IgY antibodies from egg yolk. The average recovery of venom-specific IgY from 131 eggs was about 18.47% and 5.88 g of pure IgY was obtained (Table 1). The anti-*D. acutus* IgY antibodies generated were pure and specific to HRP-rabbit anti-chicken IgY, which was revealed by SDS-PAGE and Western blot analysis (Fig. 2). It was further confirmed by Western blot analysis that the anti-*D. acutus* IgY antibodies were able to recognize and bind to most protein components of *D. acutus* venom (Fig. 5). This suggests that the obtained IgY is a specific polyclonal antibody against *D. acutus* venom.

The immunodiffusion assay indicated the anti-*D. acutus* IgY not only reacted against *D. acutus* venom, but also against *T. albolabris* and *D. saxatilis* venoms. However, it did not recognize *B. multicinctus* or *N. atra* venoms (Fig. 4). The results indicated that venoms of *D. acutus, T. albolabris* and *D. saxatilis* of the same Viperidae family possibly share some common antigen epitopes. Conversely, they did not share any antigen epitopes with venoms of *B. multicinctus* or *N. atra* of the Elapidae family. These preclinical observations will provide some reference for clinicians to use antivenom or antibodies to treat bites caused by snakes from of the same family [5, 6].

According to the proteomic analysis, there were about 128 kinds of proteins and peptides identified in the venom of *D. acutus* [31]. The Western blot analysis showed that anti-*D. acutus* venom IgY antibodies mainly recognized a molecular weight range of the protein components in the venom (including > 97.2 kDa, 66.4-29 kDa, 18–14.3 kDa), but did identify not other protein components (such as 20.1-29 kDa) (Fig. 6). Generally, protein or polypeptide components in such a mixture as crude venoms differ considerably in their abilities to elicit antibody response in immunized animals [16].

Our results indicated that protein or polypeptide components with molecular weight between 20.1 and 29 kDa in *D. acutus* venom possibly lacked some immunogenicity for chickens. According to their biochemical characterization, the identified proteins in *D. acutus* venom were divided into three groups: serine proteases; P-I class snake venom metalloproteinases (SVMPs); and other proteins [32–34]. It was reported that the serine proteases and metalloproteinases in *D. acutus* venom, those whose molecular weight was distributed in the range of > 97.2 kDa, 66.4-29 kDa and 18–14.3 kDa, contributed to the major immunogenicity of *D. acutus* venom and underpinned the hemorrhagic, edema-forming and myotoxic acitivities [31]. Our observations confirmed that the polyvalent IgY antibodies raised against *D. acutus* venom are effective in the neutralization of the most important toxic effects including lethal, hemorrhagic, edema-forming and myotoxic acitivities of *D. acutus* venom (Fig. 6, 7 and 8). In addition, the neutralization showed a characteristic dose-dependent relationship.

## Conclusion

In summary, IgY antibodies against *D. acutus* venom with high purity and titer were for the first time raised successfully in egg yolk by immunizing hens with snake venom. They were effective in neutralizing lethal, hemorrhagic, edema-forming and myotoxic acitivities of *D. acutus* venom. IgY could be an effective source to develop alternative treatment for snakebite victims in the future, either humans or other animals. However, further studies are required for testing the safety and efficacy of IgY.

## Abbreviations

CK: Creatine kinase
FCA: Freund’s complete adjuvant
FIA: Freund’s incomplete adjuvant
MED: Minimum edema-forming dose
MHD: Minimum hemorrhagic dose
MMD: Minimum myotoxic dose
OD: Optical density
PVDF: Polyvinylidene difluoride
SDS-PAGE: Sulfate-polyacrylamide gel electrophoresis
SOF: Salting-out fraction
SVMP: Snake venom metalloproteinase
TCF: Thiophilic-chromatography fraction
TMB: 3^1^,3^1^,5^1^,5^1^-tetramethylbenzidine
WSF: Water-soluble fraction.
